# CD200 Blockade Modulates Tumor Immune Microenvironment but Fails to Show Efficacy in Inhibiting Tumor Growth in a Murine Model of Melanoma

**DOI:** 10.3389/fcell.2021.739816

**Published:** 2021-10-08

**Authors:** Fatemeh Talebian, Jianyu Yu, Kimberly Lynch, Jin-Qing Liu, William E. Carson, Xue-Feng Bai

**Affiliations:** ^1^Department of Pathology, College of Medicine, The Ohio State University, Columbus, OH, United States; ^2^Division of Surgical Oncology, Department of Surgery, Comprehensive Cancer Center, The Ohio State University, Columbus, OH, United States; ^3^Comprehensive Cancer Center, Institute for Immuno-Oncology, The Ohio State University, Columbus, OH, United States

**Keywords:** CD200, CD200R, tumor immune microenvironment, cancer immunotherapy, tumor associated macrophage (TAM)

## Abstract

CD200-CD200R pathway regulates immune responses and has been implicated in the pathogenesis of a number of cancer types. CD200 blockade is considered a strategy for immunotherapy of CD200-positive cancers such as melanoma. Thus, it is critical to understand the potential impacts of CD200 blockade in a more human relevant tumor model. In this study, we evaluated these issues using the CD200^+^ Yumm1.7 mouse melanoma model. Yumm1.7 cells bear Braf/Pten mutations resembling human melanoma. We found that Yumm1.7 tumors grow significantly faster in CD200R^–/–^ mice compared to wild type mice. Analysis of tumor immune microenvironment (TIME) revealed that tumors from CD200R^–/–^ or anti-CD200 treated mice had downregulated immune cell contents and reduced TCR clonality compared to tumors from untreated wild type mice. T cells also showed impaired effector functions, as reflected by reduced numbers of IFN-γ^+^ and TNF-α^+^ T cells. Mechanistically, we found upregulation of the CCL8 gene in CD200R^–/–^ tumors. *In vitro* co-culture experiments using Yumm1.7 tumor cells with bone marrow derived macrophages (BMDM) from WT and CD200R^–/–^ mice confirmed upregulation of macrophage CCL8 in the absence of CD200-CD200R interaction. Finally, we found that anti-CD200 therapy failed to show efficacy either alone or in combination with checkpoint inhibitors such as anti-PD-1 or anti-CTLA4 in inhibiting Yumm1.7 tumor growth. Given that CD200R-deficiency or anti-CD200 treatment leads to reduced T cell responses in TME, using blockade of CD200 as an immunotherapy for cancers such as melanoma should be practiced with caution.

## Introduction

Melanoma accounts for 1% of all skin cancers, but it is the deadliest of all such cancers (Siegel et al., 2018). Melanoma is sensitive to immune modulation, partially due to the presence of tumor-infiltrating lymphocytes (TIL) in its tumor microenvironment (TME), whose presence correlates with increased survival and reduced metastasis (Candido et al., 2014; Lee et al., 2016). For decades, chemotherapy was the dominant and preferred therapeutic option for melanoma patients. However, in the past decade, we have seen major advancements in treatment options. Immune inhibitors to checkpoint molecules such as cytotoxic T-lymphocyte antigen-4 (CTLA-4) (Buchbinder and Hodi, 2016) and programmed cell death (PD-1) (Robert et al., 2019) have shown promise in treating human melanoma. However, the high rate of resistance to therapy has proven challenging. Thus, developing combination immunotherapy strategies or targeting new checkpoint molecules have gained increasing favor in overcoming resistance.

CD200 and CD200R are transmembrane glycoproteins belonging to the immunoglobulin superfamily of proteins (IgSF). CD200 contains a small 19 amino acids (AA) intracellular domain with no known signaling motif (Barclay et al., 1986). It is expressed on the surface of a wide variety of normal cells such as T and B cells, and many types of cancer cells including melanoma (Petermann et al., 2007; Liu et al., 2016; Xin et al., 2020). CD200R, its cognate ligand, is primarily expressed on myeloid cells (Wright et al., 2003). CD200R has a 67 AA cytoplasmic tail with three tyrosine residues. The 3rd tyrosine residue, located within an NPXY motif (Mihrshahi et al., 2009) inhibits phosphorylation of the ERK pathways upon ligation of CD200R to CD200 (Minas and Liversidge, 2006; Mihrshahi and Brown, 2010).

CD200R functions to control or decrease collateral damage by preventing excessive immune cell activation. Mice deficient in CD200R or CD200 are healthy, but more susceptible to induced immune pathology (Rygiel et al., 2009; Simelyte et al., 2010). The role of CD200-CD200R pathway in tumor growth, immunity and immunotherapy in mouse models is, at best, controversial. Some studies (McWhirter et al., 2006; Kretz-Rommel et al., 2007; Rygiel et al., 2012) show that CD200-CD200R interaction inhibits anti-tumor immune responses and CD200-CD200R signaling pathway plays a pro-tumor role. However, opposite results have also been reported. We previously found that B16-CD200 tumors grew significantly faster in CD200R^–/–^ mice (Liu et al., 2016). Similarly, 4THM breast tumors were shown to exhibit accelerated growth and metastasis in CD200R^–/–^ mice when compared to WT mice (Erin et al., 2015). Given that blockade of CD200 was recently used in a clinical trial for Chronic Lymphocytic Leukemia (CLL) and Multiple Myeloma (MM) (Mahadevan et al., 2019), and has been proposed for the treatment of patients with solid tumors such as melanoma, it is necessary to evaluate its potential impacts in more human relevant tumor models.

In this study, we evaluated the impact of blocking CD200-CD200R pathway in a murine model of melanoma (Yumm1.7) that bears Braf/Pten mutations (Meeth et al., 2016) resembling human melanoma. We found that blockade of CD200-CD200R either genetically or using a monoclonal antibody could significantly alter tumor immune microenvironment (TIME). We found that CD200R-deficiency resulted in significantly upregulated production of CCL8, which could partially explain why these tumors grow faster in the absence of CD200-CD200R interaction in TME. Given that CD200R-deficiency or anti-CD200 treatment leads to reduced T cell responses and fails to show benefits either alone or in combination with checkpoint inhibitors, blockade of CD200 should not be considered for immunotherapy of cancers such as melanoma.

## Materials and Methods

### Mice and Tumor Establishment

C57BL/6 mice were purchased from The Jackson laboratory, and CD200R^–/–^ mice were originally generated via a contract with Taconic Farms and were bred into the C57BL6 background for over 12 generations (Liu et al., 2016). All mice were maintained and cared for in The Ohio State University (OSU) laboratory animal facilities which are fully accredited by OSU Institutional Animal Care and Use Committee (IACUC). To establish subcutaneous (s.c.) tumors in C57BL/6 and CD200R^–/–^, 5 × 10^5^ Yumm1.7 cells in 100 μl PBS were injected into each mouse s.c. Development of tumors was monitored and tumors were measured for length (a) and width (b) every 2–3 days using a digital caliper, and tumor volumes were calculated as (a^∗^b^2^)/2.

### Nanostring Pan-Cancer Immune Profiling of Tumors

Total RNA was extracted from fresh tumors (*n* = 3/group) using Trizol^®^ according to the manufacturer’s instructions. Approximately 50 ng of total RNA were hybridized with the mouse PanCancer immune profiling code set containing 770 unique pairs of 35–50 base pair biotin-labeled capture probes and reporter probes with internal reference controls (NanoString nCounter^®^ PanCancer IO360 panel, NanoString Technologies, Inc., Seattle, WA). Hybridization was performed overnight at 65°C. Unbound probes were washed away, the tripartite structure was bound to the streptavidin-coated cartridge by the biotin capture probe, aligned by an electric current, and immobilized. Degradation of fluorophore and photobleaching were prevented by adding SlowFade. Read counts from the raw data output were assessed for differential gene expression and cell type scoring after normalization using Rosalind Software.^1^ Briefly, Log_2_ counts were represented as z-scores in heat map to indicate alterations in gene expression and immune cell profile for each sample.

### T Cell Receptor-Seq Analysis of Tumor Samples

Genomic DNA was prepared from tumor tissues and submitted for the immunoSEQ assay (Adaptive Biotechnologies, Seattle, WA). The somatically rearranged mouse T cell receptor (TCR) hypervariable complementarity-determining region 3 (CDR3) was amplified from genomic DNA of tumor samples using a two-step, amplification bias-controlled multiplex PCR approach (Robins et al., 2009; Carlson et al., 2013). Specifically, the first PCR consists of forward and reverse amplification primers specific for every V and J gene segment, and amplifies the CDR3 of the TCR locus. The second PCR adds a proprietary barcode sequence and Illumina adapter sequences. CDR3 libraries were sequenced on an Illumina instrument according to the manufacturer’s instructions. Raw Illumina sequence reads were demultiplexed according to Adaptive’s proprietary barcode sequences. Demultiplexed reads were then further processed to: remove adapter and primer sequences; identify and correct for technical errors introduced through PCR and sequencing; and remove primer dimer, germline and other contaminant sequences (Robins et al., 2012). The data is filtered and clustered using both the relative frequency ratio between similar clones and a modified nearest-neighbor algorithm, to merge closely related sequences. The resulting sequences were sufficient to allow annotation of the V(N)D(N)J genes constituting each unique CDR3 and the translation of the encoded CDR3 amino acid sequence. V, D and J gene definitions were based on annotation in accordance with the IMGT database.^2^ The set of observed TCR CDR3 sequences were normalized to correct for residual multiplex PCR amplification bias and quantified against a set of synthetic TCR CDR3 sequence analogs (Carlson et al., 2013). Data was analyzed using the immunoSEQ Analyzer toolset.

### Antibodies and Flow Cytometry

Fluorescence labeled monoclonal antibodies to mouse CD45 (30-F11), CD3 (145-2c11), CD4 (GK1.5), CD8α (53-6.7), NK1.1 (Pk136), CD11b (M1/70), Gr-1 (RB6-8C5), Ly6C (HK1.4), F4/80 (745-2342), Foxp3 (Nrrf-3c), TNF-α (XT22), IFN-γ (XMG1.2), and isotype-matched control antibodies were purchased from Biolegend or BD Biosciences. Mononuclear cells from tumors were prepared as we previously described (Liu et al., 2016). For cell surface staining, cells were incubated with antibodies in 0.1 M PBS (pH7.4) supplemented with 1% FCS and 0.1% sodium azide on ice for 30 min. Cells were then washed three times and fixed in 1% paraformaldehyde followed by flow cytometry analysis. For intracellular staining of TNF-α, IFN-γ or Foxp3, cells were first stimulated with cell stimulation cocktail (Invitrogen) for 4 h in the presence of Gorgi^*stop*^ (BD Biosciences). The cells were first stained for the cell surface markers (CD4/8), followed by a standard intracellular cytokine staining procedure. A Celesta flow cytometer (BD) was used to detect stained cells. Data was analyzed using the flowjo software (Tree Star, Inc., OR).

### Generation of Bone Marrow Derived Macrophages and Co-culture With Tumor Cells

Bone marrow (BM) cells from C57BL/6 and CD200R^–/–^ mice were plated in 15cm culture dishes in DMEM medium containing 20% FBS and 30% culture supernatant from L929-G-CSF cells. The cells were allowed to grow in an incubator at 37°C for 3 days. On day 3, fresh culture medium was added to the plate and allowed it to grow for another 2 days. Macrophages (adherent cells) were dissociated on day 5 using 5mM EDTA and re-suspended in DMEM medium containing 10% FBS and 1% L929-G-CSF supernatant. The macrophages (1 × 10^6^ cells/ml) were then co-cultured with Yumm1.7 tumor cells at 1:1 ratio or Yumm1.7 supernatant. Supernatant from another tumor cell line (NB9464D) was used as a negative control. 24 and 48 h later, culture supernatants were collected for ELISA. The detached cells were used for flow cytometry analysis or qPCR for chemokine gene expression analysis.

### ELISA

MAX CCL8 (MCP-2) ELISA kit (Biolegend) was used to quantify CCL8 in culture supernatants from BMDM/Yumm1.7 co-cultures according to manufacturer’s instructions.

### Real-Time PCR

Quantitative real-time PCR was done using previously determined conditions (33). The following primers were used for amplifying the CCL8 gene: 5′-ACGCTAGCCTCCACTCCAAA-3′ (forward) and 5′-GAGCCTTATCTGGCCCAGTC-3′ (reverse) and HPRT gene (endogenous control): 5′-AGCCTAAGATGA GCGCAAGT-3′ (forward) and 5′-TTACTACGCAGATGGCCA CA-3′ (reverse). Each sample was assayed in triplicate, and the relative gene expression was calculated by plotting the Ct (cycle number) and the average relative expression for each group was determined using the comparative method (2^–ΔΔCt^).

### Treatment of Mice With Established Tumors

We first established Yumm1.7 tumors in male or female C57BL/6 mice by injecting 5 × 10^5^ Yumm1.7 cells in 100 μl PBS. When tumors are palpable (typically 7 days after tumor cell injection), mice were treated with 250 μg/mouse of the following antibodies i.p. alone or in combination: αCD200 (OX-90), αPD1 (RPM1-14) or αCTLA-4 (9H10). Mice were treated every 3 days until the end of the experiments.

### Statistical Analyses

Statistical analyses were performed using GraphPad Prism 9.1.1 software (GraphPad Software Inc., La Jolla, CA, United States). The differences between the treatments compared to the untreated control were analyzed by multiple *t*-tests without multiple comparisons correction. The nanostring data were represented as mean of log2 fold change relative to control. All other data were presented as mean ± standard error of the mean (SEM) unless otherwise indicated. The overall *P*-value for Kaplan-Meier analysis was calculated using the log-rank test. Analysis of differences between two normally distributed test groups was performed using an unpaired *t*-test assuming unequal variance and multiple *t*-tests. *P* < 0.05 was considered to be statistically significant.

## Results

### Yumm1.7 Tumors Grow Faster in CD200R^–/–^ Mice

**FIGURE 1:**
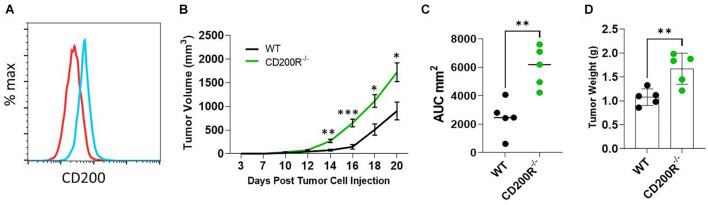
Accelerated growth of Yumm1.7 melanoma tumors in CD200R^–/–^ mice. **(A)** Flow cytometry analysis of CD200 expression in Yumm1.7 cells. Red line represents isotype antibody control, blue line represents cells stained with CD200 Antibody. **(B)** Tumor growth kinetics. Yumm1.7 cells were injected into each mouse subcutaneously at a dose of 5 × 10^5^/mouse. Tumor growth was monitored by measuring tumor length and width every 2 days after the appearance of tumors. Growth kinetics (changes of mean tumor volume over time) are shown. ^∗∗^*P* = 0.0012; ^∗∗∗^*P* = 0.00067; ^∗^*P* = 0.0101 on day 18; ^∗^*P* = 0.0017 on day 20. **(C)** Area under the curve (AUC) was calculated based on the tumor volume data shown in B (^∗∗^*P* = 0.0079). **(D)** Tumors isolated from wild type and CD200R^–/–^ mice were weighted at the end of the experiment (^∗∗^*P* = 0.0069). Data shown represents three experiments with similar results. Two sided Student’s *t*-test was used for the statistical analysis.

The Yumm1.7 melanoma tumor cell line was derived from a spontaneous tumor originally developed in a mouse bearing Braf/Pten mutations (Meeth et al., 2016). We found that Yumm1.7 cells constitutively expressed CD200 on its cell surface (Figure 1A). To investigate the role of CD200-CD200R signaling pathway in melanoma tumor establishment and progression, C57BL/6 and CD200R^–/–^ C57BL/6 mice were subcutaneously inoculated with CD200^+^ Yumm1.7 melanoma cells. About 1 week after tumor cell injection, tumors started to show up in all mice regardless of CD200R-deficiency (Figure 1B). However, tumors grew faster in CD200R^–/–^ mice compared to their wild type counterpart as measured by higher tumor growth curve (Figure 1B) and bigger area under the tumor volume curve (AUC) (Duan et al., 2012; Figure 1C). At the end of the experiment (day 20 after tumor cell injection), we sacrificed mice and measured tumor weight. We found that tumors from CD200R^–/–^ mice were significantly heavier than tumors harvested from WT mice (Figure 1D). Overall, our results show that the growth of CD200^+^ Yumm1.7 melanoma is enhanced when CD200R signaling is absent.

### Diminished Anti-tumor T Cell Responses in Yumm1.7 Tumors From CD200R^–/–^ Mice

**FIGURE 2:**
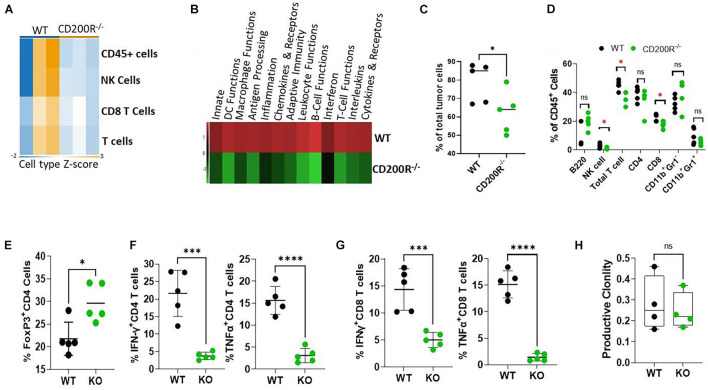
Diminished anti-tumor T cell responses in Yumm1.7 tumors from CD200R^–/–^ mice. **(A,B)** Nanostring analysis of tumors (*n* = 3/group) from WT and CD200R^–/–^ mice was performed using total tumor RNA and the Nanostring Pan cancer IO 360 panel, and results were analyzed using Rosalind software. Heat maps showing cell type abundance **(A)** in tumors and directed global significance scores **(B)** of altered pathways were generated based on Nanostring data. **(C–G)** Flow cytometry analyses of subtypes of tumor infiltrating leukocytes and effector functions of T cells. Percent of positive cells from each group was calculated relative to total tumor cells (**C**, ^∗^*P* = 0.046) or total CD45^+^ cells (**D**, ^∗^*P* < 0.05). Intracellular staining was performed to quantify Tregs (**E**, ^∗^*P* = 0.013), IFN-γ (^∗∗∗^*P* = 0.0003) and TNF-α (^****^P < 0.0001) producing CD4^+^ **(F)** or IFN-γ (^∗∗∗^P = 0.0009) and TNF-α (^****^P < 0.0001) producing CD8^+^ **(G)** T cells. **(H)** TCR-seq was performed on genomic DNA samples (*n* = 4/group) from tumors grown in WT and CD200R^–/–^ mice. Results were analyzed using ImmunoSEQ software. Depicted is productive clonality (higher values in this graph represent more clonal T cell response). Ns: not significant. Two sided Student’s *t*-test was used for all the statistical analyses.

To understand what factors were driving the enhanced Yumm1.7 tumor growth in CD200R^–/–^ mice, RNA samples from tumors were used for Nanostring analysis. We used the pre-formatted Pan-cancer 360 IO panel to characterize and measure the expression of about 750 vital genes in the TME. We first compared Immune cell type abundance within the two TME. As shown in Figure 2A, Nanostring analysis revealed decreased immune cell presence in the CD200R^–/–^ TME. In 3 out of 3 CD200R^–/–^ tumor samples, the abundance scores of immune cells were low, while 2 out of 3 tumor samples from WT mice exhibited higher enrichment of immune cells, including total CD45^+^ leukocytes, total T cells and CD8^+^ T cells. Strikingly, pathway analysis revealed that majorities of immune pathways including adaptive immunity/T cell functions were downregulated (Figure 2B). To validate the immune cell type abundance data from Nanostring, we analyzed immune cell subsets using flow cytometry. As seen in Figure 2C, flow cytometry confirmed significant decreases of CD45^+^ cells in tumors from CD200R^–^*^/^*^–^ mice. Additionally, we observed decreased percentages of NK cells, T cells (CD3^+^) and CD8^+^ T cells among total leukocytes (CD45^+^) in tumors from CD200R^–^*^/^*^–^ mice, while percentages of B cells (B220^+^) and CD4^+^ T cells among total leukocytes were not significantly different between tumors from CD200R^–^*^/^*^–^ and WT mice. Similarly, we did not detect differences in CD11b^+^Gr1^–^ tumor associated macrophage (TAM) population and CD11b^+^Gr1^+^ myeloid derived suppressor (MDSC) cells (Figure 2D). CD4^+^FoxP3^+^ regulatory T cells (Tregs) are significant players in regulating T cell responses in TME. We therefore quantified Tregs and found that Tregs were significantly increased in the CD200R^–^*^/^*^–^ tumors (Figure 2E). To determine whether the functions of immune cells such as T cells were impacted by the lack of the CD200-CD200R interaction, we quantified IFN-γ and TNF-α levels in CD4^+^ and CD8^+^ tumor infiltrating lymphocytes (TIL). We found that both IFN-γ and TNF-α production were significantly suppressed in CD4^+^ (Figure 2F) and CD8^+^ T cells (Figure 2G) within the CD200R^–^*^/^*^–^ tumors.

To assess the impact of CD200-CD200R signaling on the diversity of the immune repertoire of tumor-infiltrating T cells, we prepared genomic DNA from tumors (*n* = 4/group) and performed TCR-seq analysis (Adaptive Technology, Seattle, Washington). We found that in the absence of CD200R signaling, clonality of tumor infiltrating T cells were similar (Figure 2H), suggesting that lack of CD200R signaling did not significantly affect dominant T cell populations in TME.

### Deficiency of CD200R Signaling Alters Tumor Microenvironment and Upregulates CCL8

**FIGURE 3:**
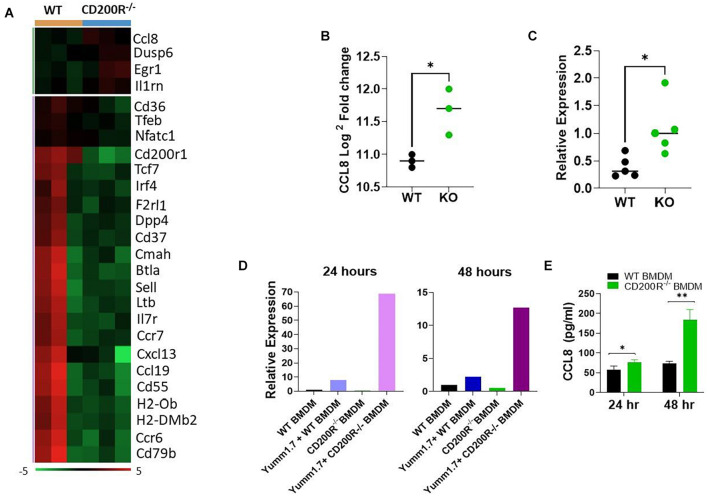
Deficiency of CD200R signaling alters TME and upregulates CCL8. **(A)** Heat map of all differentially expressed genes based on the Nanostring analysis. **(B)** CCL8 gene expression was significantly elevated in CD200R^–/–^ vs. wild type tumors based on the Nanostring analysis (^∗^*P* = 0.022 by two-sided Student’s *t*-test). **(C)** qPCR was used to quantify CCL8 gene expression in tumors (*n* = 5/group) from wild type vs. CD200R^–/–^ mice. ^∗^*P* = 0.042 by two-sided STUDENT’s *t*-test. **(D,E)** Bone marrow derive macrophages (BMDM) was generated and co-cultured with YUMM1.7 tumor cells. qPCR was used to quantify CCL8 gene **(D)** expression in macrophages, and ELISA was used to quantify CCL8 protein **(E)** production in culture supernatants. CCL8 gene expression or protein production in BMDM alone or YUMM1.7 tumor cell alone were low or undetectable. ^∗^*P* = 0.05; ^∗∗^*P* = 0.0019 by two-sided Student’s *t*-test.

Differential gene expression analysis of the data generated from the Nanaostring assay revealed significant alterations in TME (Figure 3A). Among the 26 differentially expressed genes, 22 were genes downregulated in the CD200R^–/–^ TME. Consistent with the downregulated immune pathways indicated in Figure 2A, the downregulated genes included genes important for T cell trafficking and effector functions. Notably, several chemokine and chemokine receptors (CCR6, CCR7, CCL19, CXCL13) were also downregulated. On the other end of the spectrum, there were 4 upregulated genes in TME in the absence of CD200R. Notable among them was CCL8, a chemokine ligand for murine CCR8 (Figure 3B). Our qPCR analysis verified its upregulation in tumors from CD200R-dificient mice (Figure 3C).

To determine if tumor CD200 interacts with CD200R on macrophages to regulate CCL8 production, we generated bone marrow derived macrophages (BMDM) from CD200R^–/–^ and WT mice, co-cultured them with Yumm1.7 tumor cells and measured CCL8 gene expression and protein secretion. As shown in Figure 3D, we found that CCL8 gene expression was significantly higher in CD200R^–/–^ macrophages after a 24 and 48 h co-culture with Yumm1.7 cells. At the protein level, most significant up-regulation of CCL8 was detected 48 h after co-culture (Figure 3E). Overall, these results indicate that tumor CD200 interacts with CD200R and limits the secretion of CCL8.

### CD200 Blockade Using a Monoclonal Antibody Alters Tumor Immune Microenvironment Without Inhibiting Tumor Growth

**FIGURE 4:**
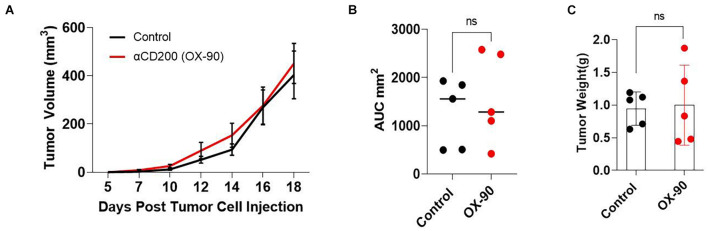
CD200 blockade using a monoclonal antibody failed to inhibit tumor growth. **(A)** Tumor growth kinetics. Yumm1.7 cells were injected into each mouse s.c. at a dose of 5 × 10^5^/mouse. Mice were treated with anti-CD200 antibody (OX-90) at a dose of 250 μg/mouse or vehicle i.p. every 3 days when tumors were palpable. Tumor growth was monitored by measuring tumor length and width every 2 days. Growth kinetics (changes of tumor volume over time) of individual tumors are shown. **(B)** Area under the curve (AUC) was calculated based on the tumor volume data shown in **(A)**. **(C)** Tumors isolated from OX-90 and vehicle treated mice were weighted at the end of the experiment. Data shown represents three experiments with similar results. Ns: not significant by two sided Student’s *t*-test.

It has been reported (Kretz-Rommel et al., 2007; Choueiry et al., 2020) that CD200 blockade may improve the efficacy of cancer immunotherapy. To determine if CD200 blockade could inhibit the growth of CD200^+^ melanoma, we tested a monoclonal antibody (OX-90) in the Yumm1.7 tumor model. OX-90 has been shown to block CD200-CD200R interaction (Snelgrove et al., 2008; Choueiry et al., 2020). We inoculated C57BL/6 mice with 0.5 × 10^6^ Yumm1.7 melanoma cells subcutaneously. Once the tumors were established, the mice were injected with the OX-90 antibody (250 μg/mouse) i.p. every 3 day until the end of the experiment. As shown in Figure 4, treatment of mice with OX-90 failed to affect Yumm1.7 tumor growth, as measured by tumor growth kinetics (Figure 4A), mean area under curve (Figure 4B) and tumor weight at the end of the experiment (Figure 4C).

**FIGURE 5:**
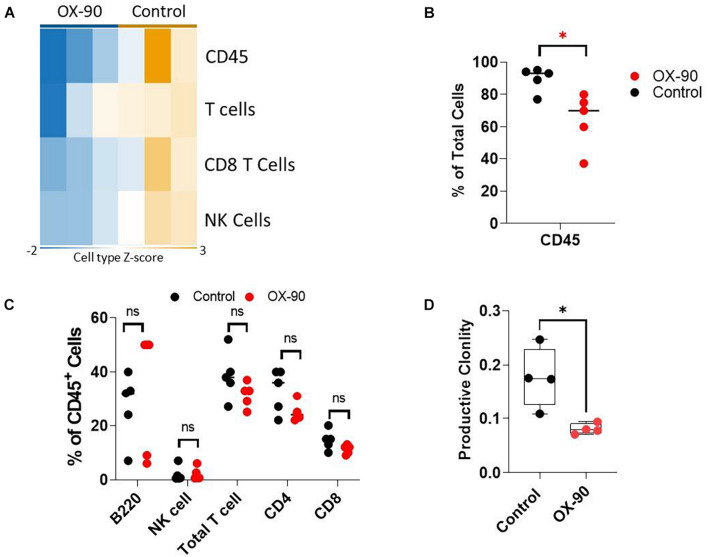
CD200 blockade alters tumor immune microenvironment. **(A)** Nanostring analysis of tumors (*n* = 3/group) from OX-90 or vehicle treated mice was performed using total tumor RNA and the Nanostring Pan cancer IO 360 panel, and results were analyzed using Rosalind software. Heat map showing cell type abundance in tumors are presented. **(B,C)** Flow cytometry analyses of subtypes of tumor infiltrating leukocytes. Percent of positive cells from each group was calculated relative to total tumor cells **(B)** or total CD45^+^ cells **(C)**. ^∗^*P* = 0.016 by two-sided Student’s *t*-test. ns: not significant. **(D)** Productive clonality of tumor infiltrating T cells. TCR-seq was performed on genomic DNA samples (*n* = 4/group) from tumors grown in OX-90 or vehicle treated mice. Results were analyzed using ImmunoSEQ software. ^∗^*P* = 0.0154 by two-sided Student’s *t*-test.

To understand if OX-90 treatment alters TIME, we analyzed the immune cell abundance using Nanostring assay. The OX-90 treated group followed a pattern similar to that seen in the CD200R^–/–^ mice. Specifically, we found reduction of total CD45^+^ cells, total T cells, CD8^+^ T cells and NK cells in tumors from OX-90 treated mice (Figure 5A). Consistent with this observation, our flow cytometry analysis verified that total CD45^+^ and T cells were reduced (Figure 5B), while proportions of other lymphocyte subsets were not significantly altered by OX-90 treatment (Figure 5C). Strikingly, ImmunoSEQ analysis indicated that OX-90 treatment decreased the clonality of tumor infiltrating T cells (Figure 5D), suggesting that OX-90 treatment decreases the quality of T cell responses in TME.

### Anti-CD200 Therapy Failed to Show Synergy With Checkpoint Inhibitors

**FIGURE 6:**
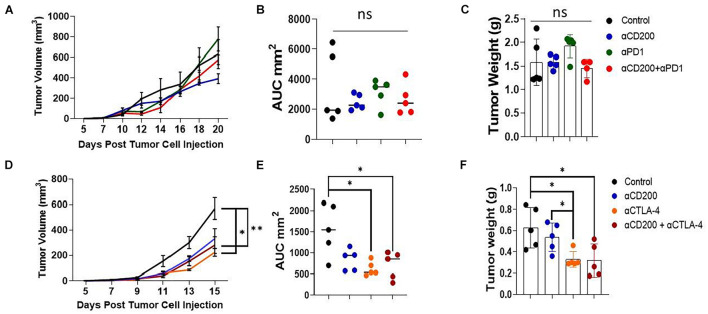
Anti-CD200 therapy failed to show synergy with checkpoint inhibitors. Yumm1.7 cells were injected into each mouse s.c. at a dose of 5 × 10^5^/mouse. When tumors were palpable, mice (*n* = 5/group) were treated with 250 μg/mouse of anti-CD200 (OX-90), anti-PD-1 (RPM1-14) or αCTLA-4 (9H10) or vehicle i.p. every 3 days. Tumor growth was monitored by measuring tumor length and width every 2 days. **(A–C)** Anti-CD200 therapy failed to show synergy with anti-PD-1 therapy. Growth kinetics **(A)**, AUC **(B)** and tumor weight **(C)** are shown. Ns: not significant by one way ANOVA. **(D–F)** Anti-CD200 therapy failed to show synergy with anti-CTLA4 therapy. Growth kinetics (**D**, ^∗^*P* = 0.02; ^∗∗^*P* = 0.005), AUC (**E**, ^∗^*P* = 0.011, control vs. aCTLA4; ^∗^*P* = 0.026, control vs. aCD200/CTLA4) and tumor weight (**F**, ^∗^*P* = 0.0115, control vs. aCTLA4; ^∗^*P* = 0.023, control vs. aCD200/CTLA4; ^∗^*P* = 0.015, aCD200 vs. aCTLA4) are shown. Data shown represents two to three experiments with similar results. One way ANOVA was used for the comparison.

Since anti-CD200 monotherapy failed to show efficacy in inhibiting Yumm1.7 tumor growth, we tested if anti-CD200 in combination with other checkpoint inhibitors will result in tumor growth inhibition. We first tested if anti-CD200 treatment had synergy with anti-PD-1 therapy. As shown in the upper panel of Figure 6, neither anti-CD200 or anti-PD-1 monotherapy, nor their combination showed any efficacy, as measured by tumor growth kinetics (Figure 6A), AUC (Figure 6B) or tumor weight at the end of the experiment (Figure 6C). Since we observed increased Tregs in TME of CD200R-deficient tumors (Figure 2D), we also examined if anti-CD200 could inhibit tumor growth in combination with anti-CTLA4 antibody 9H10. 9H10 has been shown to deplete Tregs and inhibits tumor growth (Simpson et al., 2013). We found that anti-CTLA4 antibody alone significantly reduced tumor growth as measured by tumor growth kinetics (Figure 6D), AUC (Figure 6E) or tumor weight at the end of the experiment (Figure 6F). Although OX-90 + αCTLA4 also showed significant efficacy, this combination did not prove to be more effective than αCTLA4 monotherapy. Thus, anti-CD200 therapy failed to show synergy with checkpoint inhibitors.

## Discussion

In this study, we used the human-relevant, CD200 expressing Yumm1.7 tumor model to evaluate the role of CD200-CD200R pathway in melanoma tumor growth, immunity and immunotherapy. We found that in CD200R^–/–^ mice, Yumm1.7 tumors grew significantly faster than in wild type mice. Consistent with this observation, nanostring analysis revealed that tumors from CD200R^–/–^ mice had downregulated immune cell contents. Flow cytometry analysis further suggested that Yumm1.7 tumors from CD200R^–/–^ mice had reduced infiltration of CD8^+^ T cells but increased Tregs. T cells also showed impaired effector functions, as reflected by reduced numbers of IFN-γ^+^ and TNF-α^+^ T cells. These observations are consistent with our previous observations in B16 melanoma model where CD200 was artificially expressed in B16.F10 cells (Liu et al., 2016). Similarly, 4THM breast tumors were shown to exhibit accelerated growth and metastasis in CD200R^–/–^ mice compared to WT mice (Erin et al., 2015). On the opposite end of the spectrum, it was observed that CD200^–/–^ mice had reduced carcinogen-induced tumor development (Rygiel et al., 2012). In CD200R-deficient mice, decreased growth and metastasis of CD200-positive EMT6 tumors were observed (Podnos et al., 2012). We recently provided a model (Liu et al., 2020) to explain the differential tumor growth in CD200/CD200R-deficient mice among different tumor models. Expansion of M2 macrophages and MDSCs will enhance tumor-associated inflammation/angiogenesis, activating tumor invasion/metastasis, and regulating tumor-specific T cell responses, therefore enhancing tumor growth. In contrast, expansion of M1 macrophages will lead to tumor growth inhibition due to direct anti-tumor effects imposed by M1 macrophages and induction of tumor-specific T cell responses. In this work, we found that M2 associated genes such as Il1rn (Sun et al., 2015) are significantly upregulated in TME of CD200R^–/–^ mice, suggesting more M2 conversion in the absence of CD200R signaling.

CD200-CD200R interaction may potentially affect production of inflammatory mediators by tumor-associated macrophages. In this work, we observed upregulation of CCL8 gene in CD200R^–/–^ tumors. *In vitro* co-culture experiments using Yumm1.7 tumor cells with BMDM from WT and CD200R^–/–^ mice confirmed upregulation of CCL8 in macrophages in the absence of CD200-CD200R interaction (Figure 3). CCL8 also known as MCP-2, was originally described as an antimicrobial protein involved in the inflammatory process (Asano et al., 2015). CCL8 has since been known to contribute to immune response by attracting monocytes, T lymphocytes, natural killer cells (NK), basophils, mast cells, and eosinophils (Farmaki et al., 2017). In tumor models, tumor CCL8 production has been shown to promote a pro-metastatic environment of cervical cancer (Chen et al., 2019). In TME, TAMs are major sources of CCL8 that support tumor cell survival (Cassetta et al., 2019) and increase invasion and stem-like characteristics of GBM cells (Zhang et al., 2020). CCL8 promotes the migration and invasion of esophageal squamous cell carcinoma as well (Zhou et al., 2018). High levels of CCL8 has been indicated in recruiting Tregs to the TME through CCR5 (Halvorsen et al., 2016). CCL8 is also involved in promoting metastasis in mice inoculated with human melanoma cell lines (Barbai et al., 2015). Several studies have implicated elevated CCL8 in breast cancer progression, metastasis and relapse free survival (RFS) (Farmaki et al., 2016; Farmaki et al., 2020; Tang et al., 2020). In light of these studies, we assume that upregulation of CCL8 in the absence of CD200R signaling plays a role in promoting tumor growth, which can partially explain the accelerated Yumm1.7 tumor growth observed in CD200R^–/–^ mice.

Mahadevan et al. (2019) published the results of their phase I clinical trial where they administered a novel recombinant humanized monoclonal antibody called Samalizumab that blocks CD200 in chronic lymphocytic leukemia (CLL) and multiple myeloma (MM) patients. In their study, Samalizumab was associated with reduced tumor burden in most advanced CLL patients and suggested further development of Samalizumab as an immune checkpoint inhibitor. However, Samalizumab did not prove effective in MM patients. In this work, we investigated whether CD200 blockade could be used as a potential therapy in melanoma. We found that anti-CD200 monotherapy failed to show efficacy in inhibiting Yumm1.7 tumor growth. Strikingly, we found that tumors from anti-CD200-treated mice also had downregulated abundance of immune cell contents and TCR clonality. Since activated immune cells upregulate CD200 expression, and in human trials anti-CD200 has been shown to deplete activated CD4 T cells (Mahadevan et al., 2019), we examined whether CD200 blockade resulted in depletion of CD200 expressing CD4 T cells. However, we did not observe any depletion of B cells or T cells (Figure 5C). Thus, our data suggests that anti-CD200 therapy alters tumor microenvironment through similar mechanisms observed in CD200R^–/–^ mice. Previous studies have suggested (Coles et al., 2015) that CD200 and PD-L1 should be targeted in combination to improve immunotherapy outcomes. However, in this work, we found that anti-CD200 Ab failed to show synergy with anti-PD-1 or anti-CTLA4 therapy. Thus, CD200 blockade failed to show efficacy in inhibiting Yumm1.7 tumor growth either alone or in combination with checkpoint inhibitors.

Taken together, we have found that blockade of CD200-CD200R either genetically or using a monoclonal antibody could significantly alter TIME. We found that CD200R-deficiency resulted in significantly upregulated production of CCL8, which could partially explain why tumor grows faster in the absence of CD200-CD200R interaction in TME. Given that CD200R-deficiency or anti-CD200 treatment leads to reduced T cell responses and fails to show benefits either alone or in combination with checkpoint inhibitors, blockade of CD200 should not be considered for immunotherapy of cancers such as melanoma.

## Data Availability Statement

The data has been deposited and can be accessed by using either the URL https://clients.adaptivebiotech.com/pub/talebian-2021-fcdb or 10.21417/FT2021FCDB.

## Ethics Statement

All mice works involved in this study were approved by Institutional Animal Care and Use Committee (IACUC) of the Ohio State University.

## Author Contributions

FT performed most of the experiments. JY helped and performed some animal work and flow cytometry analysis. KL and J-QL helped with mouse work and reagents. WC helped with experimental design and provided resources for the experiments. X-FB generated funding support, designed experiments, analyzed data and wrote the manuscript. All authors contributed to the article and approved the submitted version.

## Conflict of Interest

The authors declare that the research was conducted in the absence of any commercial or financial relationships that could be construed as a potential conflict of interest.

## Publisher’s Note

All claims expressed in this article are solely those of the authors and do not necessarily represent those of their affiliated organizations, or those of the publisher, the editors and the reviewers. Any product that may be evaluated in this article, or claim that may be made by its manufacturer, is not guaranteed or endorsed by the publisher.

## Footnotes

1
www.Rosalind.com

2
www.imgt.org
